# Impact of Teaching Palliative Psychology with Psychodrama and Arts Therapies on Psychology Students in Klagenfurt

**DOI:** 10.3390/bs13110931

**Published:** 2023-11-15

**Authors:** Michael Alexander Wieser, Alexandra Leitner

**Affiliations:** Department of Psychology, University of Klagenfurt, 9020 Klagenfurt am Wörthersee, Austria; aleleitn@edu.aau.at

**Keywords:** palliative psychology, arts therapies, psychodrama, students, teaching, effects

## Abstract

The Erasmus+ project “Death Education for Palliative Psychology” (DE4PP) under the coordination of the University of Padua investigated the effects of teaching palliative psychology with psychodrama and arts therapies, as positive effects on students’ attitudes towards life and death were suspected through the use of these forms of treatment. Five countries participated in this project funded by the European Commission (Austria, Israel, Italy, Poland, and Romania). In Klagenfurt (Austria), 34 students from the University of Klagenfurt completed the pilot course entitled “Palliative Psychology” generated by the project partners. Course participants filled out psychological and satisfaction questionnaires at the beginning and end of the course, to measure the impact of teaching palliative psychology with psychodrama and arts therapies. The research involved a control group. In addition, six participants in the course participated in a focus group interview at the end, which included questions about their experience of the course; the impact of arts therapies and psychodrama techniques; experiences of verbal and artistic processing of death anxiety; and the meaning of life and death, representation of death, and associated feelings. The results, in summary, showed that processing of life and death had occurred in the participants due to the course they had completed. As part of the course, creative arts and psychodrama were bridges to death. Filling out satisfaction questionnaires, photovoice assignments, role reversal, and visualizing a personal social atom were seen by students in Klagenfurt as particularly effective methods for self-reflection.

## 1. Introduction

Dempsey [1] said that dying is inevitable but distress with death is not. The subject area of palliative psychology and care is growing steadily and is also receiving increasing attention in research [2]. As a subfield of psychology, it connects all areas of palliative conditions and aims to maintain the best possible quality of life at the end of life [3,4]. Profound training of palliative psychologists is a prerequisite for the well-being of all involved, but significant deficits were identified based on recent studies [5]. It stands to reason that a palliative psychologist should both understand the needs of a dying person and be able to deal with their fear of death [1].

A confrontation with death, loss, and dying is what Testoni [6] refers to as “mortality salience” and may foster an acceptance of inevitable death. Psychodrama and arts therapies can be brought to bear to reduce the negative feelings usually associated with death [7].

Psychodrama was described by Jakob Levi Moreno (1889–1974) as a method of a psychotherapeutic nature, with its origins in child role-play, improvisational theatre, and sociometry. The basis of psychodrama is found in spontaneity, creativity, and action [8]. Accordingly, a psychodramatic session focuses on the “encounter” between people and on mapping lives and ideas onto scenes [9]. “[T]he method aims to change interactions, to free blocked spontaneity and constricted lives from outdated “cultural preserves” (Moreno). Fixations to repressed conflict situations and entrenched roles are to be set in motion” [9]. This form of therapy provides a reasonable basis for trainees in health professions to best consider clients’ well-being and respond spontaneously to obstacles. They benefit from the advantages of creative therapies in theory and practice [5].

Arts therapies include “visual/plastic arts therapy, drama therapy, dance movement therapy, music therapy, and poetry/biblio therapy” [5]. Implementing creative arts therapy in palliative care is vital [10]. Arts therapists who have received training demonstrate notable creativity and proficiency in facilitating expressive processes, which can benefit their clients’ quality of life and overall well-being [11,12,13,14,15,16].

The Erasmus+ project “Death Education for Palliative Psychology”, coordinated by the University of Padua in Italy, spanned three years (2019–2022). Its primary objective was to identify the essential knowledge and skills university students need to acquire to thrive in palliative psychology [17]. This content consisted primarily of palliative psychology, psychodrama, and arts therapies, as they specifically build on psychological strategies, coping with anxiety, and self-reflection. During the DE4PP project, a curriculum was jointly developed in all five partner countries, focusing on psychodrama and arts therapy techniques. This content was taught to interested university students as a pilot course. In our needs assessment study, Orkibi et al. [5] found high interest and confidence among students in palliative care and a deficit in training opportunities in all participating European countries.

The primary aim of this study was to find out what effects arts therapy activities and psychodrama had on university students on the course. In particular, the effects of the pilot course on students’ perceptions of death and the effectiveness of the methods introduced were considered, as well as changes in interest in the subject area of palliative care. In this article, the results from Klagenfurt are presented.

Psychodrama and arts therapy are of growing psychological and practical interest but need to be studied in health profession education, especially in palliative care and palliative psychology. The main aim of the explanatory sequential mixed-methods study presented here was to determine the impact of teaching palliative psychology with psychodrama and arts therapy. In particular, the effect of the pilot course on students’ perceptions of death and the effectiveness of the methods introduced, as well as changes in interest in the subject area of palliative care, were investigated. Positive effects on students’ attitudes toward life and death through psychodrama and arts therapies modalities were hypothesized. The data collection was both qualitative and quantitative and yielded informative results. This could lead to a change in the curricula of psychology and arts therapies programs.

## 2. Method

### 2.1. Research Design Overview

In the current study, an explanatory sequential mixed-methods approach was employed to assess the effects of incorporating psychodrama and arts therapies in the teaching of palliative psychology [18]. With this analytical and interpretative approach, we attempted to obtain a deeper understanding of the topics of death and the anxieties around it. For this purpose, a curriculum was created for interested students and taught as a pilot course. Thirty-four psychology students from the Alpen-Adria-Universität Klagenfurt participated in this course. They received six ECTS (European Credit Transfer and Accumulation System) points and a certificate for positive completion. The curriculum of this pilot course provided for the following training foci:

- Training on death and loss
- Palliative care: where, when, how
- Communication in palliative care
- Advanced care planning (ACP)
- Psychological interventions in palliative care
- Additional module: euthanasia in Austria
- Phototherapy in death education, grief processing, and dealing with continuing bonds
- Psychodrama, social atom, and death
- Intermodal arts therapy with bereaved adults
- Psychodrama for self-care of caregivers

The course consisted of nine sessions, including four workshops with practical arts therapy activities (psychodrama, phototherapy, intermodal arts therapies) totaling 20 h, with nine modules consisting of recorded videos and live video sessions. The course was offered at the Alpen-Adria-Universität Klagenfurt in the summer semester of 2021, from 8 March 2021 to 17 May 2021.

After each theoretical video, students could complete a short knowledge quiz on the respective module to earn points for successful completion and check their knowledge.

Before the start and after the course, participants were asked to complete questionnaires online (pretest and posttest). Likewise, a control group of students without treatment received these questionnaires online. Furthermore, course participants were asked to fill out a satisfaction questionnaire regarding the course online after the third and the eighth sessions. Psychological and satisfaction questionnaires were used to measure the impact of teaching palliative psychology with psychodrama and arts therapies. At the end of the pilot course, 12 participants were randomly selected to participate in a focus group interview for a final evaluation. The strengths of the focus group were that the group dynamics is part of the process, like it was in the course room. The pilot course requirements were regular attendance to the live video sessions, completion of the knowledge quizzes on each module, and a photovoice project as a final assignment.

The students saw the photovoice project as a particularly effective activity. Here, the students had to form groups, which consisted of five people. Every participant in the group was requested to take a photograph related to a topic discussed during the course and provide a concise caption comprised of a maximum of 12 words. The group members were asked to select the best photo for each group. After choosing the picture for each group, group members had to revise and change the caption for the image chosen, to reflect the group’s wishes. Next, each group had to create a Word file, no longer than a page and a half, with the first and last names of each group member, the selected photo with the caption, and a brief text describing what the image represents, why it was selected, and what theme it is associated with.

**Figure 1:**
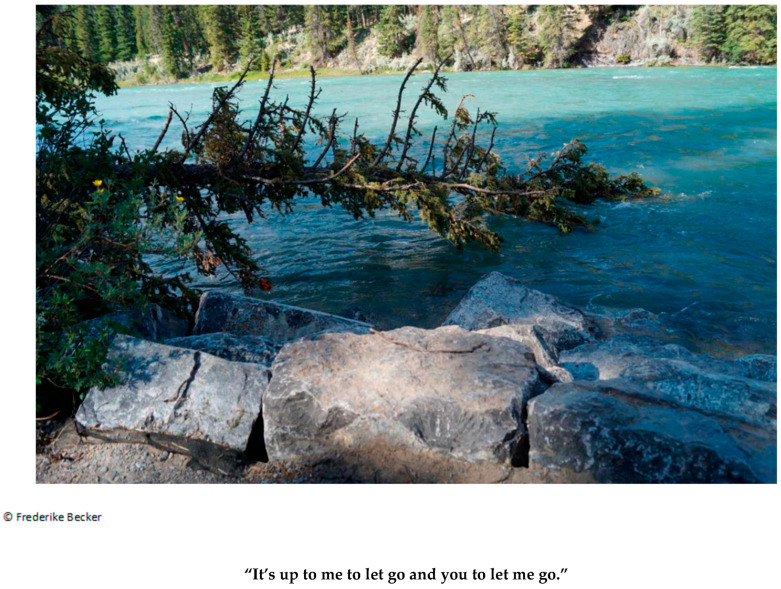
Winning photo from Klagenfurt of the photovoice task by Frederike Becker titled “It’s up to me to let go and you to let me go”.

The students found that this assignment stimulated self-reflection, as well as being enriching. The authors selected the group that performed the best photo presentation activity on a course topic (Figure 1) to go on a trip to Bologna to the ANT (Assistenza Nazionale Tumore) headquarters. There, the winning group from each country performed the following activities:

- Discussion on the issues of death and end-of-life and the activities of ANT for assistance to terminally ill people
- Role-playing activity that dealt with the theme of the end of life care
- Accompanying the ANT team in providing home care to terminally ill patients, being able to observe how they work practically in the field
- Creation of a poster that will be presented in the final dissemination meeting

After completing the course, the project partners analyzed the quantitative and qualitative findings and wrote articles. The online course is now accessible to the public at no cost. It is thus available for future use and helps recognize the significance of engaging with mortality as a valuable opportunity for personal growth and development (course documentation, http://de4pp.psy.unipd.it/2023/06/09/course-documentation-deu/, accessed on 11 September 2023). The ethics committees approved the project for the participating universities. Participating students were given consent forms to sign for each activity at the beginning of the course. Withdrawal from the pilot course and research was possible at any time and without negative consequences.

An intervention explanatory sequential mixed-methods design was used to explain the quantitative survey results with qualitative interviews.

### 2.2. Participants and Procedure

All participants were master psychology students at the Alpen-Adria-Universität Klagenfurt (Austria). A total of 64 students completed the first questionnaire, 40 of whom were from the experimental group and 24 from the control group. A total of 50 students participated in answering both the pre- and the post-questionnaire; 30 of them were from the experimental group and 20 from the control group. Thirty-four participated in the course and six in the focus group.

Of the total sample of N = 64, most students (50) were female. Fourteen indicated “male” as their gender, and no person chose the category “diverse”. The average age in Klagenfurt of those who participated was 28. Looking at religious affiliation, 49% described themselves as “Christian”, 2% as “Jewish”, one person (2%) indicated the category “Other”, and 47% did not feel they belonged to any religion. On a scale of 1 to 4, the degree of religiosity of Klagenfurt students was 2.33.

Among the survey participants, 94% reported not being involved in providing formal care for palliative patients. When asked about their personal experience of losing someone in the past two years, 63% responded negatively, while 37% answered affirmatively. In addition, 48% of the surveyed students from Klagenfurt were in the first year of their Master’s program, 27% in the second, and 25% in the third year. More detailed data can be found in Table 1.

Table 1 displays the range, mean, and standard deviation for continuous variables and the frequency and percentage for nominal variables. The final column presents the *p*-value indicating group differences, calculated using a *t*-test for continuous variables and a Chi-square test for nominal variables.

As qualitative sources, we used written open-ended answers at the end of the satisfaction questionnaires and a video focus group interview.

### 2.3. Researcher Description

The first author is a psychodrama practitioner and educator certified by the Austrian Ministry of Health, a psychologist, an assistant professor, and the course instructor. He graded the participants. The second author is now a doctoral student in psychology and the “tutor” of the course.

### 2.4. Recruitment

The students in the experimental group were interested students recruited to the university course of the professor involved in the DE4PP project and who freely chose to participate in the online course of the DE4PP project; non-participation in the research did not entail any disadvantage. They received 6 ECTS credit points and a certificate. The control group participants were enlisted by sending emails to all remaining psychology master students at the university, and they could freely choose to participate in the research. Furthermore, upon completion of the pilot course, a random number generator was used to select 12 students from the experimental group for a comprehensive focus group interview that lasted several hours. In Klagenfurt, 6 of the 12 selected could be interviewed; the other 6 were prevented by pandemic or scheduling constraints, but five 5 answered the questions in written form. The winning photovoice group of five students was sponsored for a week at the mobile hospice in Bologna.

### 2.5. Data Collection

#### 2.5.1. Quantitative Data Collection

The quantitative data were gathered through an online questionnaire created using the survey software Qualtrics, which required approximately 20 min to complete. In the case of the experimental group, the questionnaire links were provided on the E-learning platform Moodle, with one link shared at the beginning of the course and another at its conclusion. As for the control group, pretest T1 and posttest T2 questionnaires were emailed to master’s students in psychology at Alpen-Adria-Universität Klagenfurt who did not participate in the course. The experimental and control groups filled out the questionnaire simultaneously. In addition, the experimental group completed a knowledge quiz on the platform after each theoretical video.

#### 2.5.2. Qualitative Data Collection

We used written open-ended answers at the end of the satisfaction questionnaires. The qualitative survey was conducted using a focus group interview lasting several hours. The course tutor interviewed six students from the experimental group, who were randomly selected for this purpose. The interview was conducted online via the platform “Zoom”. The entire session was audio and video recorded.

### 2.6. Instruments

#### 2.6.1. Quantitative Measurement

The online questionnaire “Pretest T1 and Posttest T2” included the following demographic questions: age, gender, religion, degree of religiosity, whether formal end-of-life care is provided, whether one has lost someone in the last two years, and year of master’s degree program.

We used the following scales:

The Testoni Death Representation Scale (TDRS) [6]
The Death Attitude Profile- Revised (DAPR) [19]
The Career Commitment Scale (CCS) [20]
The Creative Self-Efficacy Scale (CSE) [21]
The Frommelt Attitude Toward Care of the Dying Scale-Form B. (FATCOD) [22]
The Compassion Scale (CS) [23]

The scales we used in this special issue were previously described in detail here [17].

#### 2.6.2. Qualitative Study

##### Focus Group Interview

To conduct a well-founded final evaluation within a small, closed group without time pressure, 12 course participants were randomly selected and asked to participate in a focus group interview. In Klagenfurt, 6 of the 12 selected could be interviewed; the other 6 were prevented by the pandemic or scheduling issues, but 5 answered the questions in written form. The interview was conducted remotely via video conference and audio and video recording. The interview duration, which encompassed breaks, was approximately three hours. The session yielded significant and captivating insights. The focus group discussion was transcribed after the interview and subjected to thematic analysis and Interpretative Phenomenological Analysis (IPA) for further examination.

The main themes of the focus group interview were as follows:

- Experiences with the course (general theme)
- The impact of arts therapies and psychodrama techniques
- The experience of verbal and artistic processing of death anxiety
- The meaning of life and death/representation of death and the feelings associated with it
- Experience of the focus group

##### Satisfaction questionnaires 1 and 2 (quantitative and qualitative)

The course participants filled out a satisfaction questionnaire online after the third and eighth sessions. To evaluate the arts-based activities of the course, the following questions were asked: How satisfied were you with

- The clarity of how to implement and deliver the arts-based activities taught in the course to patients at the end of life;
- Clarity of the theoretical basis of the arts-based activities taught in the course;
- The variation in the different arts-based activities provided in the course;
- The examples and illustrations of how the arts-based activities can be communicated to patients at the end of life;
- The relevance of the arts-based activities to your future work (as a psychologist/health care provider/psychotherapist);
- The experiential workshop with arts-based activities.

Participants could choose from six items from “very dissatisfied” to “completely satisfied”.

Furthermore, the satisfaction questionnaires asked how much they agreed with certain statements:

- The arts-based activities I have learned in this course are a valuable contribution to my future work as a psychologist/health care provider/psychotherapist;
- The arts-based activities I have learned in this course are a valuable contribution to my personal development;
- I would recommend this course to colleagues who want to learn how to use arts-based activities with patients at the end of their lives;

Here, the respondents could choose on a scale from “do not agree at all” to “agree completely”.

The satisfaction questionnaires also included general questions and suggestions, which could be answered freely in an empty text field:

- What arts-based activities from the course did you find most relevant to your future work as a psychologist/health care provider/psychotherapist?
- What kind of unclear information regarding the arts-based activities, if any, did you find in the course?
- In what ways, if any, did the arts-based activities help you develop or improve your professional skills?
- What would you recommend we add to the arts-based activities?
- What final recommendations and suggestions would you give us to improve this course?

All tests, questionnaires, and interviews were provided in German.

#### 2.6.3. Implementation Matrix

Table 2 shows the sequence of quantitative and qualitative investigations.

#### 2.6.4. Recording and Transforming the Data

The satisfaction questionnaires and knowledge quizzes were administered using Moodle’s e-learning platform. The open-ended answers were listed in a table. The psychological tests were distributed through the survey software Qualtrics. We transcribed the video of the focus group and collected the written answers of another five course participants.

### 2.7. Data Analysis

#### 2.7.1. Quantitative Data Analysis

At the outset, differences between the experimental and control group were assessed in terms of demographic variables (age, gender, religion, religious affiliation, and year of master’s degree), previous experience (as formal end-of-life caregivers and whether they had lost a loved one in the last two years), and baseline values of the study variables. Then, a 2 (time: time1 and time2) × 2 (group: experimental and control) ANOVA was performed to assess the change over time for each construct studied in the two groups. Similarly, we analyzed the time effect within each group using a *t*-test, applying the Bonferroni correction to control for the overall type I error across the two comparisons. Moreover, Cohen’s d was employed as an indicator of the effect size, where d = 0.20 represents a small effect, d = 0.50 indicates a medium effect, and d = 0.80 signifies a strong effect of time within each group. The power analysis revealed that the sample size was adequate to detect a medium effect size rather than a small-to-medium effect size of time within each group, with values of 0.98 and 0.79 in the experimental group and 0.96 and 0.73 in the control group, respectively. The sample size was somewhat problematic, detecting a small effect of time in both groups. The power was 0.35 in the experimental group and 0.31 in the control group.

#### 2.7.2. Qualitative Data Analysis

Regarding the qualitative data, 34 people from Klagenfurt participated in the survey using satisfaction questionnaires 1 and 2. The open-ended questions in the satisfaction questionnaires were evaluated using thematic content analysis. This involved analyzing the themes in the responses and subsequently comparing the results from satisfaction questionnaire 1 and satisfaction questionnaire 2. The evaluation of the satisfaction questionnaires aimed to evaluate the course with a view to future projects and course implementation.

In the focus group, of the twelve randomly selected course participants in Klagenfurt, six were interviewed, and five gave written answers. In preparation for the interview, the following research questions were formulated:

- General question: How did the students perceive the course on death, and what impact did the arts therapies and psychodrama techniques have on them?
- Specific question: How did the students experience the verbal and artistic processing of the fear of death through the arts therapies and psychodrama techniques, and what significance did they attach to them?

The aim was to explore in depth students’ perceptions of the impact of arts therapies and psychodramatic methods in the process of death education and to explore students’ experiences of verbal and artistic processing of death anxiety and its effects on them. The interviews were conducted in German, the national language.

Through the focus group interview, it was possible to answer the research questions comprehensively. Overall, a focused reflection of the whole course and a well-rounded conclusion for the participants emerged. Addressing obstacles, making suggestions for improvement, and offering comments within the focus group were possible.

The text data were analyzed using a qualitative-reflexive thematic analysis and IPA (interpretative phenomenological analysis). This approach is a theoretically flexible method for identifying key patterns of meanings and concepts throughout a dataset [24]. No presuppositions are made in this method, which is well suited for exploring participants’ experiences and perceptions [24]. The computer software QCAmap [25] was used for qualitative data analysis. All text files were uploaded to the QCAmap software and then analyzed by moving through the following stages:

1. Verbatim transcription of the focus group interview, including descriptions of participants’ tone, pitch, emotions, gestures, and group dynamics, as relevant;
2. Giving “voice” to participants’ experiences (Stage I of data analysis):
  - Development of emerging themes, patterns;
  - Search for connections between emerging themes;
  - Reading and rereading—initial notes;
  - A master list or table of themes (matrix)—capturing the participants’ experiences/“the voice”. The analysis results were five matrices, which were subsequently collected and linked by the respective project partners;
  - Amalgamating themes and superordinating themes from all focus groups [26];
  - Identifying stand-alone/‘single’ themes and taking them into account;
  - Re-examine the context to understand if the theme holds particular significance for that participant and the relevance of it to our research question and aims, to help to decide if it should be included;
  - Checking the recurrence of superordinate themes and themes [26];
  - The recurrence of superordinate themes and themes [27] was checked at the individual participant level [28] and the focus group level [27] (this was to ensure the individual’s voice and the group’s collective voice were included, to stay close to IPA’s idiographic underpinnings, while also acknowledging the value and merit of the focus group design. Including stand-alone themes, per Tomkins and Eatough’s [27] suggestions).
3. Credibility checks [26]:
  - Discuss the development and interpretation of the themes with independent IPA researchers.
4. Organizing the superordinate themes into a hierarchy [26]:
  - Create a taxonomy of themes/Themes were ordered into a “logical sequence”.
5. Writing the report, comprising two levels [26]:
  - First, the analysis section was a substantial narrative account of all themes grounded in verbatim extracts, central to IPA, to bring to the forefront participants’ voices (the basis was the resulting common matrix);
  - Second, a separate section was devoted to making sense of the results from a theoretical position. Each theme was taken in turn, described, and linked to the existing literature [29].

## 3. Results

### 3.1. Quantitative Results

For demographic variables, the experimental and control groups were similar. For religion level, there was a significant group difference with *t*(61) = −5.13, *p* < 0.001) between the control group (*M* = 2.96, *SD* = 0.69) and the experimental group (*M* = 1.95, *SD* = 0.86). Additionally, students belonging to the experimental group exhibited higher scores on the variable of semester affiliation compared to those in the control group (*χ*^2^ (2) = 17.58, *p* < 0.001). Further demographic information is given in Table 1.

The repeated measures ANOVA revealed a nonsignificant interaction effect (interaction between time and group) for the TDRS (*F* (1.48) = 2.366, *p* = 0.131). Similarly, no significant time effect was found for this scale (*F* (1.48) = 0.048; *p* = 0.827). Thus, students’ conceptions of death as a transition or extinction did not change significantly on this scale between the two measurement time points. Furthermore, no significant difference was found between the experimental and control groups (*F* (1,48) = 0.435; *p* = 0.513).

The interaction effect for the death attitude profile-revised—fear of death (DAPR) was not significant (*F* (1,48) = 2.016; *p* = 0.162). Similarly, it was found that fear of death did not change significantly between the groups (experimental and control) on this measure (*F* (1,48) = 0.80, *p* = 0.779). In addition, no significant difference was found between the two test time points on this scale (*F* (1,48) = 1.769; *p* = 0.190).

For death attitude profile-revised (DAPR)—death avoidance, no significant interaction effect for group*time was found in Klagenfurt (*F* (1,48) = 0.018; *p* = 0.895). Likewise, there were no significant differences across groups (*F* (1,48) = 0.913; *p* = 0.344) and measurement time points (*F* (1,48) = 0.440; *p* = 0.510).

For death attitude profile-revised (DAPR)—neutral acceptance, there was no interaction effect for group*time (*F* (1,48) = 3.814; *p* = 0.057). For this measure, the representations of neutral acceptance also did not change significantly between the two measurement time points (*F* (1,48) = 1.622; *p* = 0.209). No significant group differences were found between the two groups (experimental and control) either (*F* (1,48) = 0.082; *p* = 0.776).

For the career commitment scale (CCS), there was no significant interaction effect for group*time (*F* (1,48) = 0.150; *p* = 0.700). Career commitment and vocation, explicitly modified for end-of-life care, did not change significantly on this measure between the two measurement time points (*F* (1,48) = 0.275; *p* = 0.602). Similarly, no significant difference was measured between the experimental and control groups (*F* (1,48) = 0.238; *p* = 0.628).

**Figure 2:**
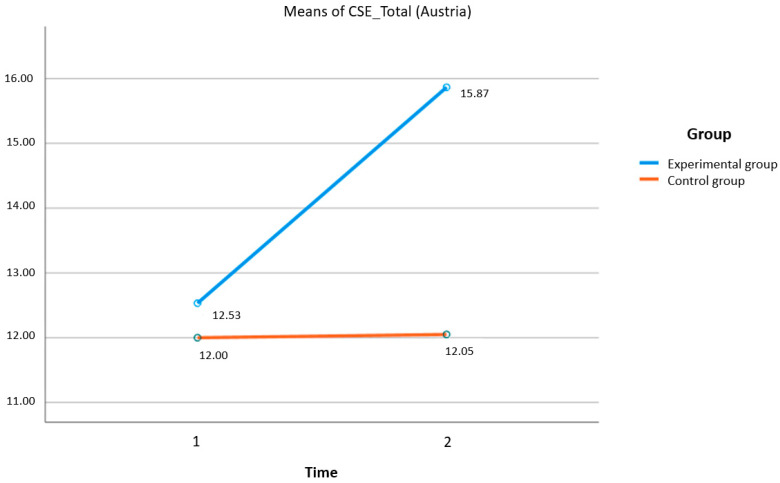
Creative self-efficacy scale (CSE).

A significant interaction effect between group and time was found on the creative self-efficacy (CSE) scale (*F* (1,48) = 7.904; *p* = 0.007, Figure 2). Participants’ self-ratings of their imagination and perceived competence in developing novel and adaptive ideas, solutions, and behaviors significantly changed for the experimental group on this scale. Similarly, the surveys showed significant main effects of time (Time 1 to Time 2) on the creative self-efficacy scale (CSE) measures (*F* (1,48) = 8.393; *p* = 0.006). In contrast, between the two groups (experimental and control), the calculations showed no significance (*F* (1,48) = 3.792; *p* = 0.057).

The compassion scale (CS) greater kindness had a positive tendency level, with *F* (1,48) = 4.037; *p* = 0.050. The items were reverse-coded. Thus, the representations of neutral acceptance, compassion—especially kindness—, and attitudes of medical and psychological staff toward the care of dying patients changed almost significantly among the students surveyed. For this scale, no significant interaction effects of group and time (*F* (1,48) = 1.317; *p* = 0.257) and no significant main impacts were found among groups (*F* (1,48) = 0.018; *p* = 0.895).

On the Frommelt Attitude Toward Care of the Dying (FATCOD) Scale—Form B, students from Klagenfurt showed no significant interaction effect of group*time (*F* (1,48) = 0.997; *p* = 0.323). In addition, no group differences could be found (*F* (1,48) = 1.801; *p* = 0.186). However, a significant difference was found between this scale’s two measurement time points (*F* (1,48) = 7.174; *p* = 0.010). There was a significant change in attitudes from participants regarding the care of dying patients.

Further values of changes over time in the experimental and control groups can be found in Table 3.

### 3.2. Qualitative Results

#### 3.2.1. Satisfaction Questionnaires 1 and 2

After the theoretical sessions and workshops, all course participants were asked to fill out a satisfaction questionnaire online, which had been prepared jointly by the project team beforehand.

The following data were collected for Klagenfurt: Thirty-four students completed the questionnaires. The average rating for satisfaction questionnaire 1 was 4.36 on a scale of 0 to 5, which means that participants were delighted with the course after the third session.

Based on the satisfaction questionnaire 2, it was possible to survey a minimal deficit in satisfaction among the students during the eighth session and, at the same time, the penultimate unit of the course. On a scale of 0 to 5, the students’ average satisfaction score was now 3.97, compared to the average score of 4.36 after the third unit.

Reasons for this included higher expectations for course content, a lack of in-depth topics, and a need for examples of practical applications.

Although there was a decrease in scores between satisfaction questionnaires 1 and 2, the course still generated high satisfaction among the participants.

#### 3.2.2. Focus Group Interview

At the end of the pilot course, a focus group interview was conducted with randomly selected students. Analyzing the data using thematic and interpretative phenomenological analysis (IPA) resulted in the following theme matrix for Klagenfurt, shown in Table 4:

Klagenfurt’s main themes can be grouped into three focus areas: positive experiences and sensations, negative experiences and sensations, and the focus group. For example, the students in the focus group found terror management theory engaging, an exciting addition, and essential input for them. One course participant said (all translations from German by A. L.):

*“I think, if that should be possible somehow, to integrate Sheldon Solomon somehow into this project in general, I think that would be really cool because I think he, um, is also really intoxicating in his lecture, and I found that somehow a very, exciting addition to the contents that we have had so far. And also, somehow really, really important what he has presented so far”.*

Similarly, they found the course they completed interesting, practical, significant, diverse, and new. In a positive sense, the course raised questions and made them think. The cooperation with Erasmus+ was noted as positive by the students. One female focus group participant summed up the issue as follows:

*“It is probably super cool to cooperate with different universities”.*

As negative experiences and perceptions, the students’ lack of willingness and motivation was criticized by the participants of the focus group interview. One participant told us the following:

*“I thought it was a bit of a pity that really so few people, um, took part because I thought that you could tell that Mr NAME had made an enormous effort, and, um, in the end, it was always quite the same people who somehow said something and thought that we were already quite a lot of people and if everyone had contributed something and if it was just a thought then the whole thing might have been a bit more fluid. In the end”; “I found it tough, sometimes, especially at the beginning, because we also got caught up with organisational things, and, uh, also phases in between, uh, which was also due to the participation of us participants, that somehow, from some the willingness or motivation was not so, there, or that, yes, they did not feel comfortable in the setting so”.*

Furthermore, the online setting was felt to be inappropriate for the topic area of palliative psychology, but this was also due to the pandemic. Likewise, a lack of conversation techniques, exercises, and practical examples was criticized, and a more in-depth knowledge transfer would have been desirable for the students. This is what one student told us:

*“I would have liked it to be a bit more interactive because if there is, in the end, an area in psychology that is really very interactive afterwards, then it is probably palliative psychology, and for that, I found it a bit too, (exhales) yes—I can’t think of the right word right now. So, I would have liked it to be more interactive, a bit more conversation techniques, et cetera”.*

Another suggestion for improvement was that the lectures should have been held in English instead of German, involving each partner country, to make the knowledge transfer more exciting and interactive.

The focus group participants positively highlighted the focus group interview itself. It was described as a perfectly rounded conclusion, and it was considered very helpful to hear the contributions of others, to stimulate thoughts. Verbalizing the ideas helped summarize everything better. Moreover, participants expressed enjoyment in discussing the course once again. One participant also highlighted the positive aspect of hearing about others’ experiences and their progress in the course. The focus group interview was summarized as a “cool experience”. One female focus group participant said:

*“I also found it really good to have been forced to reflect again on everything a little bit (laughs) the contents, uh, I think that one, or I at least, um, generally takes too little time to reflect on contents again, afterwards for oneself or in exchange with others. Um, sometimes you’re also just happy when you can then check off and then finished (laughs), um, insofar I found that actually a cool experience and, yes, I would also like it if that would be done in other seminars, so thank you, and also to the group.”*

## 4. Discussion

This study employed a mixed-methods approach to investigating the impact of incorporating psychodrama and arts therapies in teaching palliative psychology. Testoni [6] refers to confronting death, loss, and dying as “mortality salience” and argues that inevitable death should be accepted. To support this process, psychodrama and arts therapies can reduce negative feelings usually associated with death. In the present study, a change in attitude toward life and death was evident in students after completing the Death Education for Palliative Psychology (DE4PP) pilot course. Self-reflection, self-care, and an awareness of the inevitable death of any person were successfully elicited in participating students.

As the qualitative data suggest, the course qualitatively helped students to be less afraid of death, to avoid it less, and to accept it as a natural occurrence. It also increased their desire to work in palliative care and end-of-life care. It provided an opportunity for personal and professional growth, by addressing existential issues such as death and life. The psychodrama and arts therapies group workshops proved effective in processing negative emotions related to death and helped students become more confident and interested in working in this field. Completion of satisfaction questionnaires, photovoice presentations, role reversal, and visualization of a personal social atom were considered by the students to be particularly effective methods for self-reflection.

Quantitatively, their imagination and perceived competence in developing novel and adaptive ideas, solutions, behavior, and creative self-efficacy for end-of-life care changed significantly, as did their compassion, understood as kindness, a sense of humanity, mindfulness, and attention to the suffering of others. Likewise, the attitudes of course participants to the care of dying patients improved.

The experimental group was in a higher semester than the control group. This was due to the selection criteria for advanced courses. Forty-five students applied, but the university`s maximum course size for advanced classes is 35. First, we took 40, but five students soon found out they would need more time resources and quit. To handle the missing data, we set 35 “as treated”.

Some students found it confusing in the satisfaction questionnaires that the scale starts with “Very dissatisfied” and asked us to correct their entry because they thought it was “very satisfied”, but we could not change that.

All course materials are available, to show examples of arts-based techniques that can facilitate work in health-based professions (De4PP—Death Education for Palliative Psychology (unipd.it), http://de4pp.psy.unipd.it/, accessed on 11 September 2023). Profound training of palliative psychologists is a prerequisite for the well-being of all involved [5]. Therefore, it is crucial to prioritize the integration of palliative psychology in health professions education. This will not only enable healthcare professionals to assist those in need but also to maintain their well-being. While grappling with death can be challenging, individuals across all professions should be equipped to address their fears and concerns surrounding mortality [1]. The DE4PP project achieved positive results and partially achieved acceptance of life and death among the participants.

## 5. Limitations and Future Directions

Limitations in the study include bias due to social desirability, which may have been reflected in the group interviews and questionnaire responses. The researchers were also the course instructors and gave the grades. These roles should be separated. It would be beneficial to contrast data from instructors of the pilot course and student perceptions. Smaller group sizes in parallel classes should be aimed for in the future, as well as scheduling more time for practical exercises. Some of the students were opposed to online teaching. Nevertheless, if informed consent is received and online education is replicated, we will also more thoroughly analyze video recordings of the live meetings and the chats during the course.

The sample showed an imbalance in gender, as most participants were female, but this is the usual case with psychology students. Future studies could be conducted to examine the impact of the end-of-life care course on other randomly selected students, evenly distributed for gender and extended to students from other health professions who may be associated with or come into contact with the field of end-of-life care.

The control group needed more reminders at T1 and T2 to reach the minimum of 20 participants. However, the time difference between T1 and T2 was, on average, the same as in the experimental group.

Another limitation was the sample size. This would need to be expanded to arrive at more generalizable results, especially in the focus group.

An advantage of future studies would be to conduct individual interviews with all course participants, as several results and insights could be collected through intensive conversations. This study shown an increased need for palliative care and health profession education, especially in psychology programs, and that this need should be met soon. A future study should also include a comparison group, besides an experimental and control group. The comparison group should receive the same benefits as the experimental group.

## Figures and Tables

**Table 1:** Descriptive Statistics for Demographic Variables by Group.

		Experimental Group (N = 40)	Control Group (N = 24)	Group Difference *p*-Value
Age:		21–39; 27.51 (6.57)	22–51; 29.21 (6.89)	0.928
Gender:				0.876
	Male	9 (23%)	5 (21%)	
	Female	31 (77%)	19 (79%)	
	Other	0 (0%)	0 (0%)	
Religion:				0.518
	Christian	19 (49%)	12 (50%)	
	Jew	1 (2%)	0 (0%)	
	Buddhist	0 (0%)	1 (4%)	
	None	19 (49%)	11 (46%)	
	Religious level	1–4; 1.95 (0.86)	1–4; 2.96 (0.69)	<0.001
Formal caregiver to end-of-life-clients:				0.494
	No	37 (95%)	22 (92%)	
	Yes	2 (5%)	2 (8%)	
Lost someone close to you in the last two years:				0.554
	No	25 (64%)	15 (63%)	
	Yes	14 (36%)	9 (37%)	
Year of master’s degree:				<0.001
	1st	26 (67%)	5 (21%)	
	2nd	10 (26%)	7 (29%)	
	3rd	3 (7%)	12 (50%)	

**Table 2:** Implementation Matrix.

Session	Date	Module	Format
1	8 March 2021	Introduction and Pretest of measures (T1) Training on death and loss Palliative care: Where, when, how	1.5 h live online session Recorded Recorded
2	15 March 2021	Communication in Palliative Care Advanced Care Planning	Recorded Recorded
3	22 March 2021	Psychological Intervention in Palliative Care Euthanasia in Austria Satisfaction assessment-I	Recorded Recorded
4	12 April 2021	Phototherapy in Death Education, Grief Processing, and dealing with Continuing Bonds + explanation of final photovoice assignment	Recorded + 1 h live online session
5	19 April 2021	Psychodrama, Social Atom, and Death	Recorded + 3 h live online session
6	26 April 2021	Intermodal Arts Therapy with Bereaved Adults	Recorded + 3 h live online session
7	3 May 2021	Psychodrama for Self-Care of Caregivers	Recorded + 3 h live online session
8	10 May 2021	Completion Satisfaction assessment-II Posttest of measures (T2)	1.5 h live online session
9	17 May 2021	Focus Group Interview	3 h live online session with selected students

**Table 3:** Descriptive measurements of both groups at both time points.

Experimental Group (N = 30)							Control Group (N = 20)					
Variable	Time 1		Time 2		Time Effect		Time 1		Time 2		Time Effect	
	M	SD	M	SD	t	Cohen’s d	M	SD	M	SD	t	Cohen’s d
TDRS total	20.13	6.32	19.27	5.92	1.18	0.22	18.30	5.74	18.95	5.34	–1.35	–0.30
DAPR Fear of Death	2.96	0.90	2.74	0.86	2.00	0.37	2.78	0.79	2.79	0.79	–0.07	–0.02
DAPR Death Avoidance	2.19	0.94	2.23	0.76	–0.35	–0.06	1.96	0.74	2.02	0.83	–0.88	–0.20
DAPR Neutral Acceptance	4.13	0.45	4.17	0.52	–0.50	–0.09	4.21	0.54	4.02	0.52	2.41	0.54
DAPR Approach Acceptance	2.49	0.76	2.53	0.75	–0.41	–0.07	2.57	0.83	2.61	0.67	–0.35	–0.08
DAPR Escape Acceptance	2.94	0.96	3.01	0.83	–0.73	–0.13	2.85	0.92	3.03	0.84	–1.21	–0.27
CCS Total	12.57	3.52	12.90	3.59	–0.60	–0.11	12.20	4.15	12.25	4.24	–1.17	–0.04
CSE Total	12.53	3.78	15.86	4.52	–4.79	–0.88	12.00	4.35	12.05	4.95	–0.05	–0.01
CS Greater Kindness	16.07	1.77	15.90	1.92	0.87	0.16	16.37	2.11	15.74	1.91	1.61	0.37
CS Lesser Indifference	7.41	1.90	7.31	2.22	0.28	0.05	8.37	2.01	8.16	2.24	0.31	0.07
FATCOD Total	3.96	0.36	4.11	0.40	–3.04	–0.57	3.86	0.35	3.93	0.43	–1.02	–0.23

**Table 4:** Matrix of Themes in Klagenfurt using Interpretative Phenomenological Analysis (IPA). N = 6.

Theme	Subtheme
1. Positive experiences and sensations	1.1. Terror management theory was a rousing, exciting addition and important input 1.2. The course was interesting, practical, important, diverse, and new 1.3. The course raised questions in a positive sense and was thought-provoking 1.4. The lecturer put in an insane amount of effort 1.5. Anticipation for more was awakened 1.6. Erasmus’s cooperation
2. Negative experiences and sensations	2.1. Lack of willingness and motivation of students 2.2. Online setting was inappropriate 2.3. Contents were partly too theoretical and boring 2.4. Missing conversation techniques and exercises, as well as practical examples 2.5. Topics were mostly treated superficially; more depth would have been desirable 2.6. Lectures in English from partner countries would have been more exciting and interactive
3. Focus Group	3.1. Was a perfectly rounded conclusion 3.2. It was very helpful to hear the contributions of the others to stimulate thoughts 3.3. By verbalising the thoughts, everything could be summarised better 3.4. It was fun to talk about it again 3.5. It was nice to hear how the others are doing and how they have been with the course 3.6. It was good to reflect on everything again 3.7. Cool experience

## Data Availability

The data supporting this study’s findings are available at https://researchdata.cab.unipd.it/790/ (accessed on 11 September 2023).
